# Adaption and Degradation Strategies of Methylotrophic 1,4-Dioxane Degrading Strain *Xanthobacter* sp. YN2 Revealed by Transcriptome-Scale Analysis

**DOI:** 10.3390/ijms221910435

**Published:** 2021-09-28

**Authors:** Yingning Wang, Fang Ma, Jixian Yang, Haijuan Guo, Delin Su, Lan Yu

**Affiliations:** 1 State Key Laboratory of Urban Water Resource and Environment, School of Environment, Harbin Institute of Technology, Harbin 150090, China; lina_wang2@163.com (Y.W.); yangxj@hit.edu.cn (J.Y.); sudelin717@126.com (D.S.); 15B927074@hit.edu.cn (L.Y.); 2 College of Energy and Environmental Engineering, Hebei University of Engineering, Handan 056107, China; guohaijuan@163.com

**Keywords:** adaption, biodegradation, 1,4-dioxane, methylotroph, transcriptome

## Abstract

Biodegradation of 1,4-dioxane (dioxane) contamination has gained much attention for decades. In our previous work, we isolated a highly efficient dioxane degrader, *Xanthobacter* sp. YN2, but the underlying mechanisms of its extraordinary degradation performance remained unresolved. In this study, we performed a comparative transcriptome analysis of YN2 grown on dioxane and citrate to elucidate its genetic degradation mechanism and investigated the transcriptomes of different dioxane degradation stages (T0, T24, T48). We also analyzed the transcriptional response of YN2 over time during which the carbon source switched from citrate to dioxane. The results indicate that strain YN2 was a methylotroph, which provides YN2 a major advantage as a pollutant degrader. A large number of genes involved in dioxane metabolism were constitutively expressed prior to dioxane exposure. Multiple genes related to the catabolism of each intermediate were upregulated by treatment in response to dioxane. Glyoxylate metabolism was essential during dioxane degradation by YN2, and the key intermediate glyoxylate was metabolized through three routes: glyoxylate carboligase pathway, malate synthase pathway, and anaplerotic ethylmalonyl–CoA pathway. Genes related to quorum sensing and transporters were significantly upregulated during the early stages of degradation (T0, T24) prior to dioxane depletion, while the expression of genes encoding two-component systems was significantly increased at late degradation stages (T48) when total organic carbon in the culture was exhausted. This study is the first to report the participation of genes encoding glyoxalase, as well as methylotrophic genes *xoxF* and *mox*, in dioxane metabolism. The present study reveals multiple genetic and transcriptional strategies used by YN2 to rapidly increase biomass during growth on dioxane, achieve high degradation efficiency and tolerance, and adapt to dioxane exposure quickly, which provides useful information regarding the molecular basis for efficient dioxane biodegradation.

## 1. Introduction

1,4-Dioxane (dioxane), a cyclic ether with two ether linkages, is very widely used as a solvent in industry and is common in many industrial products, as well as industrial and municipal wastewaters. Dioxane pollution in water and soil has been around for decades [1]. As a Group 2B carcinogen certificated by the International Agency for Research on Cancer (IARC), the presence of dioxane in the environment has gained much attention. Due to the special physicochemical properties of dioxane, it remains dissolved in water rather than evaporating, and it is also resistant to sorption [2]. Bioremediation has more advantages than other treatments for dioxane contamination; thus, it has become the most promising strategy to eliminate dioxane pollution [3]. However, the application of microorganisms for the bioremediation of dioxane is still limited by slow degradation kinetics [4].

Glyoxylate has been confirmed to be a key intermediate in dioxane degradation [5]. It is commonly dissimilated through the glyoxylate cycle [6]. Usually, glyoxylate is formed from isocitrate cleavage by isocitrate lyase and then combined with acetyl–CoA by malate synthase to form malate [6]. However, the metabolism of glyoxylate is quite different in methylotrophic bacteria. Methylotrophs metabolize glyoxylate through the ethylmalonyl–CoA pathway instead of or simultaneously with the glyoxylate cycle [7,8]. In the ethylmalonyl–CoA pathway, glyoxylate is converted to malate as well, but the reaction is catalyzed by another enzyme, (3S)–malyl–CoA thioesterase [9].

Methylotrophs have been found to be promising in applications for both industrial and environmental biotechnology, including the synthesis of biofuels and biofertilizers, production of value-added metabolites, and toxin bioremediation [10,11,12,13,14,15,16]. Methylotrophs have many advantages in terms of biomass productivity and metabolic efficiency [10,11,17,18,19]. In addition, some methylotrophs can be considered to be real “super-bugs” as they are tolerant of environments with extreme pH or temperature, high concentrations of metals, sulfates, and a variety of pollutants [20,21,22,23,24]. Although the mechanisms remain unknown, the special characteristics of methylotrophs have made them potential novel platforms for future biotechnologies, especially for bioremediation of contamination. 

We previously reported a novel dioxane degrader, *Xanthobacter* sp. YN2, which possessed extraordinary degradation performance, fast growth kinetics, and extremely high tolerance during the metabolism of dioxane [25]. The discovery of this strain may bring a solution to obstacles in the bioremediation of dioxane. In this study, based on genome- and RNA-sequencing results, we confirmed that this strain is a methylotroph. To our knowledge, transcriptional analysis of dioxane degradation by methylotrophs has not been reported previously. By means of a transcriptome-scale analysis of cultures growing with dioxane and/or citrate at three different degradation stages, multiple strategies used by strain YN2 to achieve high degradation performance and swift adaption to dioxane were identified, as well as several novel degradation pathways and genes. These results could provide a valuable genetic resource to further explore the molecular mechanisms of dioxane degradation, offering a blueprint and instruction to enhance the application of dioxane degraders.

## 2. Results

### 2.1. RNA-Sequencing Results and Assembly

Approximately 10.26 million 150-bp paired-end raw reads were generated for the 18 samples by RNA sequencing. After filtration for quality control, approximately 10.20 million clean reads were acquired, and 94.51% with Phred-like quality scores at the Q30 level were selected as high-quality reads for further analysis. The reads were mapped to the genome of YN2 (GenBank accession numbers CP063362-CP063366), and the mapping rates of all samples were higher than 98%. A summary of the RNA sequencing results is presented in Table 1. According to the validation of RNA sequencing results by RT-qPCR (Figure S1), the RNA-sequencing method used in this study was reliable and accurate.

**Figure 1:**
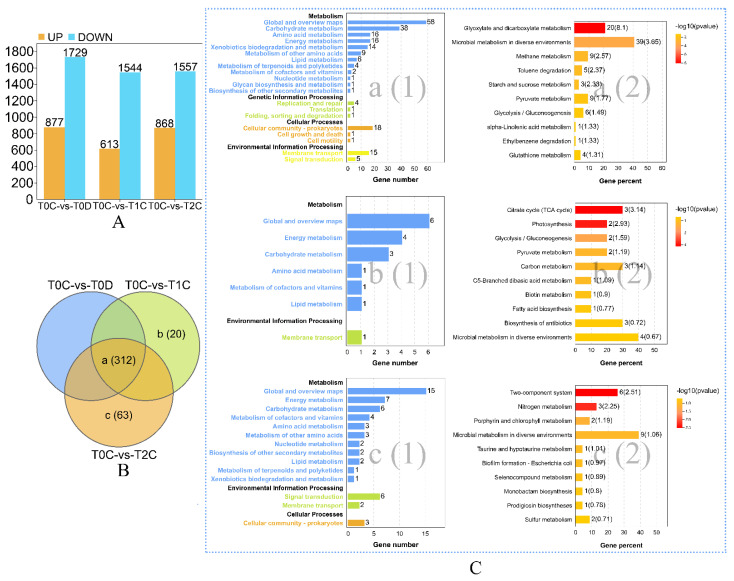
Overview of the strain YN2 transcriptome response to dioxane treatment: (**A**) number of individual transcripts significantly upregulated or downregulated of each group; (**B**) Venn diagram illustrating the classification of upregulated DEGs. Genes were filtered by FPKM ≥ 50 to remove non-essential pathways; (**C**) KEGG pathway enrichment analysis of DEGs of the three classes. Data were visualized using column diagrams. The number of DEGs mapped into each pathway is presented in figure No. (1) of each class and labeled by the end of the column. *p*-value levels of enriched pathways are presented in figure No. (2) of each class, with the number of components in each pathway present in the DEG dataset labeled by the end of the column, and indicated by “−log10 (*p* value)” and an enrichment factor indicative of individual pathways in parentheses.

Genes related to dioxane degradation in strain YN2 were studied at the transcriptional level. According to the RNA sequencing results of T0C-vs-T0D, the expression of 2606 genes of strain YN2 differed significantly during growth on dioxane relative to growth on citrate; 877 genes were upregulated with dioxane relative to the citrate control, whereas 1729 genes were downregulated (Figure 1A).

Gene regulation in response to dioxane exposure was investigated by treating citrate-grown YN2 with dioxane. After treatment with dioxane for 24 h, 2157 DEGs were found in T0C-vs-T1C; 613 DEGs were upregulated, and 1544 DEGs were downregulated. After treatment with dioxane for 48 h, 2425 DEGs in T0C-vs-T2C were found; 868 DEGs were upregulated, and 1557 DEGs were downregulated (Figure 1A). 

**Figure 2:**
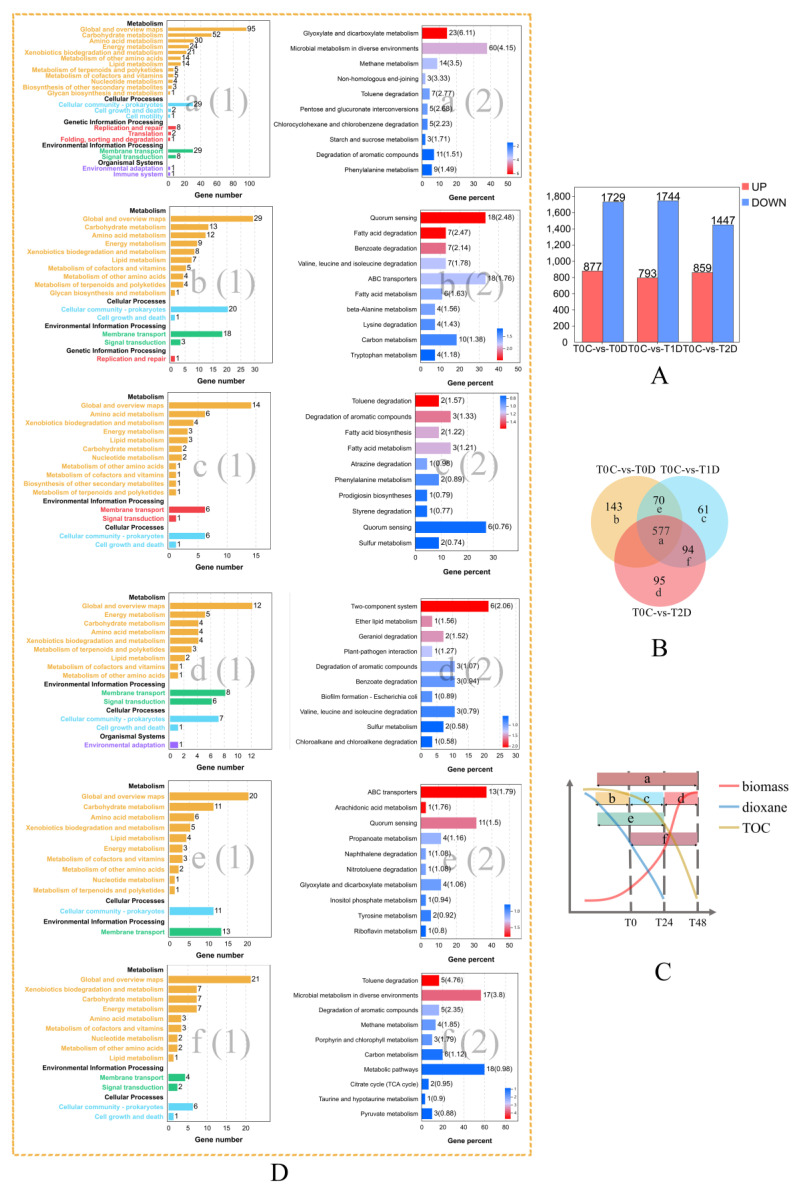
Overview of the dynamic transcriptome during three dioxane degradation stages of the strain YN2: (**A**) number of individual transcripts significantly upregulated or downregulated of each group; (**B**) Venn diagram illustrating the classification of correlations of upregulated DEGs. Genes were filtered by FPKM ≥ 50 to remove non-essential pathways; (**C**) sketch of growth and dioxane degradation curve of YN2. The red line represents a biomass of YN2, the blue line represents dioxane concentration, and the brown line represents TOC concentration. Letters correspond to correlation classes, and surrounding color blocks represent the time range of each class; (**D**) KEGG pathway enrichment analysis of DEGs of the six classes. Data were visualized using column diagrams. The number of DEGs mapped into each pathway is presented in figure No. (1) of each class and labeled by the end of the column. *p*-value levels of enriched pathways are presented in figure No. (2) of each class, with the number of components in each pathway present in the DEG dataset labeled by the end of the column and indicated by “−log10 (*p* value)” and an enrichment factor indicative of individual pathways in parentheses.

The transcriptional results of YN2 during three stages of dioxane metabolism were compared with those of YN2 grown on citrate. The numbers of DEGs at T0, T24, and T48 are presented in Figure 2A.

All the DEGs were subjected to enrichment analysis using Basic Local Alignment Search Tool (BLAST) searching against the NCBI non-redundant sequence database (NCBI Nr protein database, http://www.ncbi.nlm.nih.gov, accessed on 4 March 2020), the Kyoto Encyclopedia of Genes and Genomes (KEGG, http://www.genome.jp/kegg, accessed on 4 March 2020), and Gene Ontology (GO, http://www.geneontology.org, accessed on 4 March 2020).

### 2.2. Essential Pathways Related to Metabolism of and Response to Dioxane

To identify key pathways related to the metabolism of and response to dioxane, the relationships between upregulated DEGs of T0C-vs-T0D, T0C-vs-T1C, and T0C-vs-T2C (listed in Table S2) were analyzed, and the results are presented in Figure 1B,C (genes were filtered by FPKM ≥ 50 to remove non-essential pathways). For genes exclusively upregulated in T0C-vs-T1C, 90.00% (18/20) were annotated to KEGG, and the citrate cycle (ko00020) was of the highest representation, with 3 upregulated genes mapped to this pathway. For genes upregulated in T0C-vs-T2C alone, 61.90% (39/63) were annotated to KEGG, and two-component systems (ko02020) were at the top significance, with 6 upregulated DEGs mapped to this pathway. Among genes upregulated in all 3 groups, 24.68% (77/312) were annotated to KEGG, among which 20 genes were annotated in glyoxylate and dicarboxylate metabolism (ko00630), the most significantly enriched pathway, indicating that glyoxylate is a key intermediate during dioxane degradation by YN2.

### 2.3. Genes Involved in Metabolism from Dioxane to Glyoxylate

Soluble di-iron monooxygenases (SDIMOs) are the key enzymes that initially hydroxylate the ring of dioxane. The six components of the two SDIMOs gene clusters of YN2 (*thmABCDEF*) showed differential expression regulation throughout the metabolism of dioxane (Table 2). The gene clusters were expressed constitutively, *thmA* and *thmB* were both upregulated in all six groups, while *thmC*, *thmD*, and *thmE* were not upregulated in T0C-vs-T0D. The expression of *thmF* was not upregulated at any time during dioxane metabolism.

Genes encoding alcohol/methanol dehydrogenase were significantly upregulated during the metabolism of dioxane (Table 2). A methanol dehydrogenase gene cluster consisting of *moxR*, *moxI*, *moxG*, *moxJ*, *moxF*, *moxY*, and *moxX* was found to be involved in dioxane metabolism. This gene cluster showed low transcriptional levels when the strain YN2 was grown on citrate and was strongly induced by dioxane treatment, as shown in Table 2. On the contrary, GE01514, GE02036, GE03038, GE06014, and GE06229 encoding alcohol dehydrogenase were all expressed constitutively with extremely high transcriptional levels. GE02036, GE06014, and GE06229 were upregulated in all six groups, while GE01514 only showed upregulation at T48 (T0C-vs-T2C and T0C-vs-T2D), which suggests that this gene mainly participated in the late stage of dioxane degradation. GE03038 was upregulated in T0C-vs-T2C, T0D-vs-T1D, and T0D-vs-T2D, indicating that it may take part in degradation processes prior to GE01514. According to the reported dioxane degradation pathway, these alcohol/methanol dehydrogenases potentially participate in the transformation of 2-hydroxy-1,4-dioxane and ethylene glycol [5].

Glyoxalases were proposed to be engaged in dioxane degradation and catalyze the transformation of glyoxal to glycolate [5,26]. In the genome of strain YN2, 14 glyoxalase encoding genes were found, among which GE00986, GE04331, and GE04345 were upregulated by dioxane compared with citrate (Table 2). 

Genes encoding aldehyde dehydrogenases of strain YN2 were strongly upregulated by dioxane treatment (Table 2). Three constitutive genes encoding aldehyde dehydrogenase, GE02965, GE05991, and GE05994, were upregulated by treatment with dioxane. The transcriptional levels of these genes were very high both during citrate metabolism and throughout dioxane metabolism.

Two glycolate transformation gene clusters were found in the genome of YN2, and both were upregulated by dioxane (Table 2). The first glycolate transformation gene cluster consisted of *glcD*, *glcE*, *glcF*, and *glcB* (malate synthase encoding gene); the second glycolate transformation gene cluster contained *glcD*, *glcE*, and *glcF*. 

### 2.4. Genes Involved in Metabolism of Glyoxylate

Upregulated DEGs suggest divergent routes for glyoxylate metabolism in YN2. The genome of YN2 contained a glyoxylate degradation gene cluster, which was strongly upregulated by dioxane (Table 3). This cluster consisted of *glxR*, *hyi*, and *gcl*, which are predicted to encode tartronate semialdehyde reductase, hydroxypyruvate isomerase, and glyoxylate carboligase, respectively. In addition, gene GE05377, encoding glycerate 2-kinase (Gck) was also upregulated by dioxane, although it was distant from the glyoxylate degradation cluster, and its FPKM was much lower. Apart from glyoxylate degradation proteins, malate synthase can also catalyze the conversion of glyoxylate [27,28]. During dioxane metabolism by YN2, GE01457, encoding malate synthase, was significantly upregulated. Meanwhile, GE03599, encoding malate dehydrogenase, which catalyzes the transformation of malate to oxaloacetate, was also significantly upregulated. These findings suggest that glyoxylate metabolism through malate is essential for dioxane degradation in YN2. In *Acetobacter*, glyoxylate oxidase can convert glyoxylate to oxalate through glyoxylate oxidase [29], but genes encoding this enzyme were not found in the genome of YN2.

Genes related to the ethylmalonyl–CoA pathway were regulated differentially during the metabolism of dioxane, including genes encoding acetyl–CoA acetyltransferase, acetoacetyl–CoA reductase, 3-hydroxybutyryl–CoA dehydratase, crotonyl–CoA reductase, methylmalonyl–CoA epimerase, ethylmalonyl–CoA mutase, (2S)-methylsuccinyl–CoA dehydrogenase, (2S)-methylfumaryl–CoA hydratase, malyl–CoA lyase, propionyl–CoA carboxylase alpha chain, propionyl–CoA carboxylase beta chain, (3S)-malyl–CoA thioesterase, and methylmalonyl–CoA mutase (Table 3).

### 2.5. Dynamic Transcriptome through Three Stages of Dioxane Degradation

To understand the dynamics of the YN2 transcriptome during dioxane degradation, the whole catabolic process was divided into three stages, and transcripts at each stage were examined at T0, T24, and T48 (denoted as T0D, T1D, and T2D), and then compared with T0C. The total number of DEGs of the three groups is presented in Figure 2A. The correlations among upregulated DEGs (corresponding genes were filtered by FPKM ≥ 50 to remove non-essential pathways and listed in Table S3) are presented in Figure 2B and branched to six classes. A sketch of the growth and dioxane degradation curve of YN2, as in Figure 2C, shows the different degradation stages as well as the range of each correlation class. The degradation process was divided into three stages: the early stage, the middle stage, and the late stage. The most significant KEGG pathways associated with the entire degradation of dioxane were glyoxylate and dicarboxylate metabolism (ko00630). The most significant pathways enriched from exclusively upregulated DEGs of each degradation stage were quorum sensing (ko02024), toluene degradation (ko00623), and two-component systems (ko02020), as in Figure 2D. In addition, ATP-binding cassette (ABC) transporters (ko02010) were significantly upregulated from the early stage to the middle stage, and toluene degradation (ko00623) was significantly upregulated from the middle stage to the late stage. Since *thmABCDEF* has not been captured by KEGG, it was annotated into toluene degradation (ko00623), and the upregulation of this KEGG pathway indicated the upregulation of dioxane degradation genes.

#### 2.5.1. Genes Involved in Quorum Sensing

**Figure 3:**
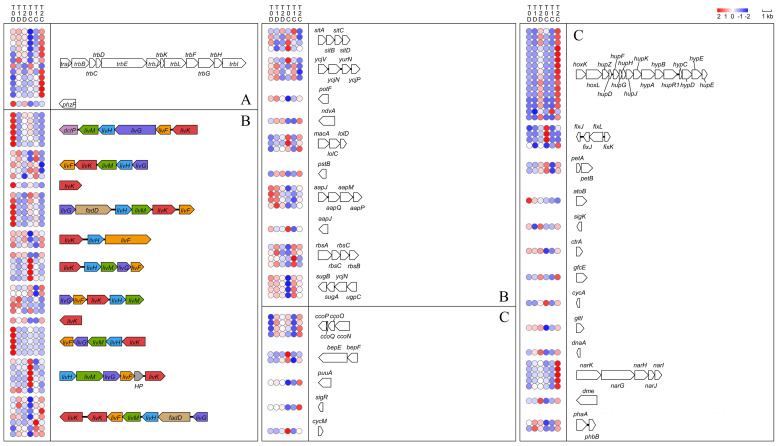
Heatmap and schemes of DEGs related to (**A**) quorum sensing, (**B**) ABC transport system, and (**C**) two-component system involved in dioxane degradation. Circles represent heatmap of gene expression, with genes ordered vertically. HP is the abbreviation for hypothetical protein.

Quorum sensing pathway genes were notably upregulated at the early degradation stage, as shown in Figure 2D. Therefore, we analyzed genes related to quorum sensing and found 362 quorum sensing-related genes, 7 of which were significantly upregulated in T0C-vs-T0D and T0C-vs-T1C (Table S4), with a *trb* gene cluster involved, as shown in Figure 3A. Research shows that the *trb* gene cluster is involved in conjugation in *Agrobacterium* strains, and the adjoining gene *traI* is responsible for the production of conjugation factor, a member of the family of substituted homoserine lactones [30]. Both Tra and Trb are part of a type IV secretion system, which transports proteins or DNA-protein complexes across cell membranes in Gram-negative bacteria [31]. It has been confirmed that some pathogens use this system to create a suitable environment for bacterial colonization [32,33]. 

#### 2.5.2. Genes Encoding Transporters

Genes encoding ATP-binding cassette transporters were significantly upregulated in both T0C-vs-T0D and T0C-vs-T1D (Figure 2D). Thus, we analyzed genes and clusters encoding ABC transporter proteins, and many of them were found to be upregulated at different stages of dioxane metabolism (Figure 3B). Related genes are listed in Table S5. As in Figure 3B, among ABC transporter genes and clusters, the number of upregulated *liv* genes and clusters was considerable. Studies have shown that the polycistronic message including *livK*, and *livHMGF* encodes the high affinity, branched-chain amino acid transport system LS for leucine and phenylalanine in *Escherichia coli* [34,35,36]. Studies have also revealed that *livK* may be related to bacterial stress response [37]. In addition, other upregulated ABC transporter genes and clusters such as *sitABCD* and *ycjV* were also reported to help bacteria resist stressful environments [38,39]. 

#### 2.5.3. Genes Encoding Two-Component Systems

Genes encoding two-component systems were significantly induced at late stages of dioxane degradation. As shown in Figure 1C and Figure 2D, two-component systems were significantly upregulated in both T0C-vs-T2D and T0C-vs-T2C. We analyzed the transcripts of genes related to two-component systems (as listed in Table S6), and the upregulated genes are presented in Figure 3C. There were two clusters that were significantly upregulated during the late stages of dioxane metabolism. One was a large cluster consisting of *hoxKL*, *hupDEFGHJKZ*, and *hypABCDE*. These genes are commonly involved in hydrogen oxidation in nitrogen-fixing aerobes [40,41,42,43,44]. The other was the gene cluster *narGHIJK* encoding respiratory membrane-bound nitrate reductase, which participates in denitrification [45]. In addition, *fixK* (nitrogen fixation) was also significantly upregulated in T0C-vs-T0D, as well as at the late stages of dioxane metabolism (T2D and T2C). *Xanthobacter* is able to fix dinitrogen under chemoheterotrophic conditions, but this activity usually happens only at reduced oxygen tension and in the absence of organic nitrogen sources or ammonia [46]. The upregulation of genes related to nitrate reductase at the late stages matches with the fact that species of *Xanthobacter* are nitrogen-fixing hydrogen bacteria [46]. However, the correlation between nitrogen fixation and dioxane degradation requires further research to reveal.

#### 2.5.4. Genes Involved in Other Important Cellular Functions

Research has confirmed that chemotaxis is an important cellular physiological response contributing to degradation [47]. In this study, we also observed the regulation of some genes involved in chemotaxis and cell motility due to dioxane metabolism. Among 22 related genes, 3 genes were upregulated in T0C-vs-T0D, 2 genes were upregulated in T0C-vs-T1C, and 1 gene was upregulated in both T2C and T2D compared with other samples. Regulated genes related to chemotaxis are listed in Table S7.

## 3. Discussion

Strain YN2 possesses better dioxane degradation performance and tolerance than many other isolates [25], which may be the result of multiple strategies in their response to dioxane. One of the strategies YN2 may use to deal with dioxane metabolism is the high expression of key genes. Previously, we reported that the genes of YN2 encoding the key enzyme SDIMOs were constitutive [25]. In this study, more genes related to dioxane degradation were found to be constitutive, and the transcription levels were already very high before dioxane treatment. In T0C-vs-T0D, FPKM reads of 64 upregulated DEGs in T0C were higher than 500, and the number of upregulated DEGs in T0C with FPKM ≥ 100 reached 210. After treatment with dioxane, these genes were further upregulated to higher levels (see details in Table S8). This strategy allows the cells of YN2 to quickly initiate efficient dioxane metabolism. 

Another strategy used by strain YN2 during the metabolism of dioxane is the use of multiple pathways for the metabolism of key intermediates. As described in the results, strain YN2 metabolized glyoxylate in three ways. Genes of the glyoxylate degradation pathway (*glxR*, *hyi*, and *gcl*) and malate synthase pathway (*glcB*) were both upregulated significantly by dioxane to extremely high expression levels (Table S8). Among reported dioxane degraders, *Pseudonocardia dioxanivorans* CB1190 is the most well studied [48]. Metabolism of glyoxylate through malate by strain CB1190 was not confirmed since the gene encoding malate synthase was not upregulated by dioxane treatment [49], while this route is clear in YN2 according to the transcriptome results. Genes encoding glyoxylate oxidase are not found in either the genome or transcriptome of YN2, indicating that oxalate is not a key intermediate during dioxane degradation, which is also the case for CB1190 [5]. The third route for glyoxylate metabolism in YN2 is the ethylmalonyl pathway, which is a unique pathway in methylotrophs [50]. The transcriptome results showed that some genes belonging to this pathway were upregulated by dioxane, while the gene encoding (3S)-malyl–CoA thioesterase, the enzyme that directly catalyzes the synthesis of malate [50], was not upregulated, which means that the ethylmalonyl pathway is probably anaplerotic during the metabolism of dioxane by YN2.

Apart from high transcription levels of related genes and various metabolism routes, strain YN2 also uses multiple genes that participate in single steps of dioxane degradation. As shown in Table 2, genes upregulated by dioxane contained two soluble di-iron monooxygenase gene clusters, five alcohol dehydrogenase genes, and one methanol dehydrogenase gene cluster, three glyoxalase genes, three aldehyde dehydrogenase genes, and two glycolate oxidase gene clusters. The number of genes involved in the conversion of dioxane to glyoxylate in YN2 was at least twice that in the strain CB1190. This multi-gene strategy benefits YN2 the same way as the two strategies mentioned above, which offers the strain more options and stronger enzyme activity to deal with dioxane metabolism, thus enhances the efficiency in terms of degradation.

The present study identified genes involved in the metabolism of dioxane and focused on gene regulation during different degradation stages and the transcriptional changes in response to dioxane exposure. As mentioned above, many genes related to dioxane degradation of strain YN2 were constitutive, which means that the genes are more “prepared” than induced ones. Meanwhile, many genes of YN2 related to quorum sensing, transporters, two-component systems, and chemotaxis were significantly upregulated in T0C-vs-T0D, T0C-vs-T1C, and T0C-vs-T2C (Figure 1C and Figure 2D). These genes are related to cell density, gene expression adjustment, and sensing, response, and adaption to changes in the environment [37,38,39,46,51,52], which helps YN2 rapidly increase biomass on dioxane and quickly adapt to dioxane exposure. The fast growth kinetics of YN2 on dioxane has been confirmed by experiments [25]. By comparison, the cell yield of the strain CB1190 is much lower than that of YN2, and genes related to quorum sensing are not upregulated by treatment with dioxane in CB1190 [25,49]. Research has shown that certain concentrations of dioxane can stimulate the bacterial generation of acyl–homoserine lactones (a quorum-sensing signal), enhancing biofilm biomass formation of the dioxane degrader *Acinetobacter baumannii* DD1 [53]. By this token, both (a) degradation genes being constitutively expressed and (b) increased expression of genes related to quorum sensing, transporters, and two-component system allow cells of YN2 to adjust efficiently to dioxane, ensuring the fast growth kinetics on dioxane, therefore aiding the rapid degradation kinetics of YN2.

Along with the four strategies in response to dioxane, being a methylotroph may also be an advantage for dioxane degradation of the strain YN2. First of all, *Xanthobacter* species are generally methylotrophic [46]. Our analysis of the genome and transcriptome showed that YN2 was isocitrate lyase negative. Isocitrate lyase-negative methylotrophs have higher efficiency of carbon recovery than isocitrate lyase-positive bacteria [17]. In addition, many methylotrophs have a high tolerance to a variety of extreme conditions, toxic pollutants of high concentration included [18,19,20,21]. This characteristic matches with the high dioxane tolerance of YN2 we reported previously [25]. Studies have shown that the high tolerance of methylotrophs may result from the expression of multiple copies of important genes, as well as several dissimilatory pathways assisting detoxification [54]. This may be also one of the strategies used by YN2 to deal with dioxane, as described above.

**Figure 4:**
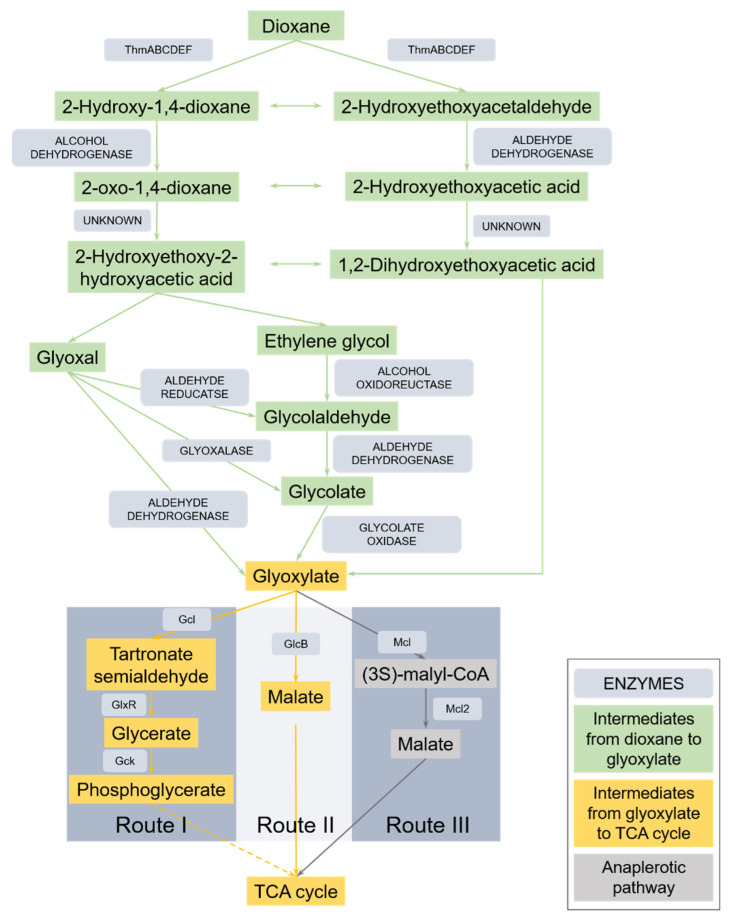
Proposed dioxane metabolic pathway in *Xanthobacter* sp. YN2 based on the results in the present study and existing research. The dashed arrow indicates multistep transformations. Gcl, glyoxylate carboligase; GlxR, tartronate semialdehyde reductase, Gck, glycerate 2-kinase; GlcB, malate synthase; Mcl, malyl–CoA lyase; Mcl2, (3S)-malyl–CoA thioesterase.

During this research, many new genes related to dioxane degradation were identified. Genes encoding glyoxalase were proposed to participate in the metabolism of glyoxal during dioxane degradation, but this speculation was not proved in CB1190 [5]. In this work, three glyoxalase-encoding genes were upregulated by dioxane (Table 2), suggesting the involvement of glyoxalase in dioxane metabolism. In addition to glyoxalase, another enzyme, methanol dehydrogenase, was also significantly upregulated by dioxane (Table 2), which to our knowledge has never been reported before. Strain YN2 has the ability to grow robustly on methanol, with a generation time shorter than on dioxane [25]. It has two types of methanol dehydrogenases, Ln^3+^-dependent XoxF and Ca^2+^-dependent MoxRIGJFYX. Genes encoding these two kinds of enzymes are very distinct because they are indicator genes of methylotrophs and generally only exist in methylotrophs [10,55]. XoxF was constitutive with high expression levels and was significantly upregulated during the late stages of dioxane metabolism; the *mox* cluster was significantly induced by dioxane, and the transcriptional levels of the whole cluster continued to increase along with the metabolism of dioxane (Table S8). This result indicated that methanol may be an intermediate during dioxane degradation of YN2. Although alcohol dehydrogenases have already been reported to play an important role in dioxane degradation and share some similarity with methanol dehydrogenases [5,56,57], the participation of these special methylotroph indicators in dioxane metabolism is a novel discovery. Dioxane degradation pathway of YN2 was proposed in Figure 4.

Gene lateral transfer may be of considerable evolutionary significance to the biodegradation of dioxane. Monooxygenases, the key enzymes that catalyze the cleavage of the ether bond of dioxane [25,58], are considered to result from gene lateral transfer during the evolution [59]. Many strategies of strain YN2 mentioned above are also abilities that may have been gained through gene lateral transfer, such as genes related to quorum sensing [60], chemotaxis [61], and two-component systems [62]. Intriguingly, gene lateral transfer is also quite evident throughout the evolution of methylotrophy, especially the indicator gene *xoxF* [10]. Studies on evolution have declared that lateral gene transfer helps microbes develop the abilities for rapid response and adaptation to environmental contamination [61,63]. Further studies are underway to show the taxonomic position and ecophysiological significance of strain YN2 and also to help reveal the role of gene lateral transfer in the evolution of dioxane degraders.

## 4. Materials and Methods

### 4.1. Chemicals and Culture Media

Dioxane was of analytical grade (J&K Scientific Ltd.: Beijing, China). Ammonium mineral salts medium (AMS) was prepared by the method of Parales et al. [48]. To limit the volatilization of dioxane, all experiments were carried out in Teflon-sealed bottles.

### 4.2. Experimental Setup

Three sets of experiments were performed to investigate the gene expression changes of strain YN2 grown on different substrates over time. All sets of experiments were carried out in duplicate and repeated three times. Analysis of dioxane and total oxidizable carbon (TOC) were carried out, as described previously [25]. 

The first set of experiments was designed to study differential gene expression (DGE) between cells grown on citrate and dioxane. For this, cells were inoculated into AMS with 5 mM citrate or 5 mM dioxane and cultivated at 30 °C with shaking at 180 r/min. Cells were harvested during the exponential phase when half of the dioxane was depleted and stored at −80 °C until RNA extraction. The RNA sequencing results for this group were designated as T0C-vs-T0D.

The second set of experiments was performed to explore gene expression during the whole degradation process. Cells were inoculated into AMS with 5 mM dioxane and samples were harvested when the growth reached the early exponential phase, and half of the substrate was depleted (denoted as T0). Samples were harvested again at 24 h when dioxane could no longer be detected in the culture (denoted as T24), and at 48 h, when TOC of the culture system ceased to decrease (denoted as T48). All cells were stored at −80 °C immediately after harvest until RNA extraction. The RNA-sequencing results of this set of experiments were designated as T0D, T1D, and T2D.

The third set of experiments aimed at investigating gene expression changes when the carbon source switched from citrate to dioxane. Cells were initially incubated in AMS with 5 mM citrate at 30 °C and shaken at 180 r/min and harvested when the growth reached the early exponential phase, and half of the substrate was depleted (denoted as T0). The pellets were washed twice with AMS and resuspended in fresh AMS amended with 5 mM dioxane and incubated as described above. Samples were harvested again at the point when dioxane was longer detected in the culture (about 24 h after resuspension, denoted as T24), and for the third time, when TOC of the system ceased to decrease (about 48 h after resuspension, denoted as T48). All cells were stored at −80 °C immediately after harvest until RNA extraction. The RNA-sequencing results of this set of experiments were designated as T0C, T1C, and T2C.

### 4.3. RNA Extraction, cDNA Library Construction, and Sequencing

Total RNA was extracted by the TRIzol-based method (Life Technologies, Carlsbad, CA, USA), followed by quality control. mRNA was enriched by removing rRNA with Illumina MRZB12424 Ribo-Zero rRNA Removal Kit (Bacteria) (Illumina, San Diego, CA, USA). The first-strand cDNA was synthesized using ProtoScript II Reverse Transcriptase. The second-strand cDNA was synthesized using NEBNext Second-Strand Synthesis Reaction Buffer and dATP, dGTP, dCTP, dUTP mix (New England BioLabs, Ipswich, MA, USA). The resulting cDNA was purified with Agencourt AMPure XP beads (Beckman Coulter, Brea, CA, USA) and end repaired with NEBNext End-Repair Reaction Buffer and Enzyme Mix (New England BioLabs). Sequencing adapters were ligated using NEBNext Adaptor for Illumina (New England BioLabs). The second-strand cDNA was then degraded using the USER Enzyme Mix (New England BioLabs), and the product was purified by Agencourt AMPure XP beads (Beckman Coulter). Finally, the sequencing library was constructed using NEBNext Poly(A) mRNA Magnetic Isolation Module (New England Biolabs).

The clustering of the index-coded samples was performed on a cBot Cluster Generation System according to the manufacturer’s instructions. After cluster generation, sequencing was performed using the Illumina Novaseq 6000 platform with pair-end 150 base reads.

Quality trimmed reads were mapped to the reference genome (GenBank accession numbers: CP063362-CP063366) using Bowtie2 [64] (version 2.2.8), allowing no mismatches. Reads that mapped to ribosomal RNA were removed. Retained reads were aligned with the reference genome using Bowtie2 [64] (version 2.2.8) to identify unknown genes. Gene expression levels were calculated by RSEM [65].

Sequence data were deposited to NCBI Sequence Read Archive (SRA) database under bioproject ID: PRJNA640796.

### 4.4. Differentially Expressed Genes Analysis

The gene expression level was normalized by using the fragments per kilobase of transcript per million mapped reads (FPKM) method to eliminate the influence of either different gene lengths or the amount of sequencing data on the calculation of gene expression. The edgeR package (http://www.r-project.org/ accessed on 4 March 2020) was used to identify differentially expressed genes (DEGs) across samples. Genes with a fold change ≥ 1.2 and a false discovery rate (FDR) < 0.05 were identified as significant DEGs, and *p* values were corrected using 0.05 as the threshold.

### 4.5. Gene Expression Validation

In total, 14 genes among the most upregulated genes, including *thmABCDEF*, GE06014, GE05990, GE02965, GE02036, GE05991, GE05994, GE02333, and GE05988, were selected for validation by quantitative real-time reverse transcriptase-PCR (RT-qPCR). RNA was extracted from samples described above using the Maxigen HiPure Total RNA Mini Kit according to the manufacturer’s instructions (including the optional DNase treatment). Reverse transcription was performed with FastQuant RT Kit (Tiangen Biotech, Beijing, China) according to the manufacturer’s instructions.

RT-qPCR analyses were performed by amplification of the cDNA samples from above using the Bestar SybrGreen qPCR Mastermix, according to the manufacturer’s instructions. Primer sequences are listed in Table S1. Thermocycling conditions were as follows: 2 min at 95 °C, followed by 45 cycles of 10 s at 95 °C, 1 min at 60 °C, followed by melting curve analysis. Expression of the 16S rRNA gene was used as the reference gene to normalize tested genes. The ΔΔCt method with the 16S rRNA gene as the reference was used to determine the relative abundance of target transcripts.

## 5. Conclusions

The highly efficient and tolerant dioxane degrader *Xanthobacter* sp. YN2 uses four genetic or transcriptional strategies to respond to dioxane exposure, achieve large biomass quickly, and maintain efficient degradation performance: (1) many dioxane degradation related genes are constitutive, and their expression levels are already very high before dioxane treatment, reducing time and stress for transcriptional response; (2) various routes (glyoxylate carboligase pathway, malate synthase pathway, and anaplerotic ethylmalonyl–CoA pathway) participate in metabolism of glyoxylate, the key dioxane degradation intermediate of YN2, increasing degradation efficiency; (3) multiple enzymes appear to be involved in individual degradation steps, therefore increasing catalytic efficiency and accelerating the degradation process; (4) when cells are exposed to dioxane, expression of quorum sensing and transporter genes increase, and when the carbon source is exhausted, upregulation of genes related to two-component systems occurs, helping the strain YN2 adapt to environmental changes rapidly. In addition, YN2 is an isocitrate lyase-negative methylotroph, which has a big advantage in pollutant biodegradation with high efficiency and tolerance. Additionally, evidence for the involvement of glyoxalase-encoding genes in dioxane degradation is reported in the present work. Furthermore, methylotrophic genes *xoxF* and *mox* are the first to be reported involved in dioxane degradation by this study. This work provides genetic and transcriptional insights into microbial adaption and response to dioxane, and it will be useful for the performance enhancement of dioxane degraders.

## Figures and Tables

**Table 1:** Sequencing and assembly statistics of YN2 transcriptome data.

Sample	^a^ T0C	T0D	T1C	T1D	T2C	T2D
Number of raw reads (×10^6^)	10.18	11.10	8.41	12.05	10.17	9.67
Number of clean reads (×10^6^)	10.15	11.07	8.39	12.02	10.15	9.65
Clean reads Q30 (%)	94.20	94.38	94.43	94.39	94.99	94.63
Clean reads ratio (%)	99.69	99.71	99.79	99.78	99.81	99.77
Mapping ratio (%)	98.43	99.17	98.93	98.65	98.90	98.66
Uniquely mapping ratio (%)	81.87	80.25	74.62	75.26	78.08	77.89

^a^ The meaning of each sample: T0C, strain YN2 grown on citrate and reaching the early exponential phase; T0D, strain YN2 grown on dioxane when half of the dioxane degraded; T1C, 24 h after carbon source switched from citrate to dioxane; T2C, 48 h after carbon source switched from citrate to dioxane; T1D, strain YN2 grown on dioxane when dioxane depleted; T2D, strain YN2 grown on dioxane when TOC (total oxidizable carbon) ceased decreasing.

**Table 2:** Genes involved in catalyzation of dioxane to glyoxylate.

Gene ID		Gene Name	Gene Description	^b^ Fold Change Compared with Citrate				
				T0D	T1C	T2C	T1D	T2D
Monooxygenase gene cluster (*thmABCDEF)*	GE02043	*thmA*	monooxygenase α subunit	^a^ **1.31**	**1.63**	**1.28**	**1.62**	**1.39**
	GE06236							
	GE02042	*thmB*	monooxygenase γ subunit	**1.23**	**1.72**	**1.40**	**1.55**	**1.28**
	GE06235							
	GE02041	*thmC*	ferredoxin	1.03	**1.44**	**1.30**	**1.24**	1.08
	GE06234							
	GE02040	*thmD*	coupling/effector protein	1.09	**1.51**	**1.36**	**1.40**	**1.24**
	GE06233							
	GE02039	*thmE*	monooxygenase β subunit	1.17	**1.64**	**1.53**	**1.64**	**1.44**
	GE06232							
	GE02038	*thmF*	ferredoxin oxidoreductase	0.75	0.92	1.05	0.89	0.87
	GE06231							
Alcohol dehydrogenase encoding genes	GE01514	*xoxF*	alcohol dehydrogenase	1.02	0.60	**2.00**	1.09	**2.49**
	GE02036	*ybdR*	alcohol dehydrogenase	**1.23**	**1.70**	**1.50**	**1.74**	**1.50**
	GE03038	*adh*	alcohol dehydrogenase	0.88	0.65	**1.71**	**1.71**	**1.41**
	GE06014	*adh*	alcohol dehydrogenase	**1.34**	**1.52**	**1.34**	**1.45**	**1.35**
	GE06229	*ybdR*	alcohol dehydrogenase	**1.23**	**1.70**	**1.50**	**1.74**	**1.50**
Methanol dehydrogenase gene cluster	GE04892	*moxR*	ATPase	**8.00**	**5.94**	**9.99**	**8.92**	**15.94**
	GE04893	*moxI*	methanol dehydrogenase	**41.95**	**34.47**	**48.91**	**63.09**	**75.12**
	GE04894	*moxG*	cytochrome c-L	**50.96**	**8.24**	**13.87**	**13.13**	**22.92**
	GE04895	*moxJ*	methanol oxidation system protein	**14.51**	**11.39**	**19.71**	**21.19**	**35.92**
	GE04896	*moxF*	methanol dehydrogenase	**79.15**	**72.69**	**108.39**	**135.63**	**169.09**
	GE04898	*moxY*	methanol utilization control sensor protein	0.78	0.43	0.92	0.74	**1.71**
	GE04899	*moxX*	methanol utilization control regulatory protein	0.96	0.76	1.27	**1.38**	**2.10**
Glyoxalase encoding genes	GE00986		glyoxalase	**5.86**	**6.84**	**8.06**	**6.21**	**6.77**
	GE04331			**2.23**	1.37	1.17	**2.45**	**1.76**
	GE04345			**1.23**	0.34	0.35	0.50	0.42
Aldehyde dehydrogenase encoding genes	GE02965	*aldA*	aldehyde dehydrogenase	**1.82**	**1.55**	**1.63**	**1.55**	**1.94**
	GE05991	*aldHT*	aldehyde dehydrogenase	**3.20**	**3.08**	**3.21**	**3.00**	**2.84**
	GE05994	*ald*	aldehyde dehydrogenase	**4.15**	**4.27**	**3.94**	**3.78**	**3.85**
Glycolate oxidase gene cluster 1	GE01453	*glcD*	glycolate oxidase subunit	**2.87**	**2.94**	**2.15**	**2.99**	**2.95**
	GE01454	*glcE*	2-hydroxy-acid oxidase	**2.55**	**2.33**	**1.81**	**2.54**	**2.34**
	GE01455	*glcF*	2-hydroxy-acid oxidase	**2.22**	**2.70**	**2.00**	**2.40**	**2.35**
	GE01457	*glcB*	malate synthase	**8.26**	**4.29**	**6.51**	**5.87**	**6.71**
Glycolate oxidase gene cluster 2	GE05986	*glcF*	2-hydroxy-acid oxidase	**1.59**	**1.99**	**1.57**	**1.32**	**1.45**
	GE05987	*glcE*	2-hydroxy-acid oxidase	**1.68**	**2.38**	**1.82**	**1.45**	**1.70**
	GE05988	*glcD*	FAD-binding protein	**2.95**	**3.40**	**2.41**	**2.53**	**2.39**

^a^ Fold change ≥ 1.2 with FDR and *p* value < 0.05 are indicated in boldface. ^b^ The meaning of each sample compared with citrate (T0C): T0D, strain YN2 grown on dioxane when half of the dioxane degraded; T1C, 24 h after carbon source switched from citrate to dioxane; T2C, 48 h after carbon source switched from citrate to dioxane; T1D, strain YN2 grown on dioxane when dioxane depleted; T2D, strain YN2 grown on dioxane when TOC ceased decreasing.

**Table 3:** Genes related to the metabolism of glyoxylate.

Gene ID	Gene Name	Gene Description	^b^ Fold Change Compared with Citrate				
			T0D	T1C	T2C	T1D	T2D
Glyoxylate degradation gene cluster							
GE02332	*glxR*	Tartronate semialdehyde reductase	^a^ **11.89**	**10.98**	**11.89**	**13.30**	**12.95**
GE02333	*hyi*	Hydroxypyruvate isomerase	**13.11**	**11.9**	**15.14**	**15.50**	**15.48**
GE02334	*gcl*	Glyoxylate carboligase	**28.46**	**21.81**	**20.80**	**27.91**	**25.94**
Other genes related to glyoxylate degradation							
GE05377	*gck*	glycerate 2-kinase	**2.59**	**1.44**	**2.35**	**2.10**	**2.27**
Gene related to the ethylmalonyl–CoA pathway							
GE05373	*phaA*	Acetyl–CoA acetyltransferase	**2.31**	**1.52**	**2.99**	**2.97**	**2.71**
GE03297			**1.26**	0.96	0.93	1.11	1.04
GE05374	*phbB*	Acetoacetyl–CoA reductase	1.16	1.06	**1.84**	**2.08**	**1.64**
GE01950	*croR*	3-Hydroxybutyryl–CoA dehydratase	**1.49**	0.95	**1.77**	**1.57**	**1.61**
GE00219	*ccr*	Crotonyl–CoA reductase	**2.13**	0.78	**1.53**	**1.44**	**1.42**
GE04789	*yqjC*	Methylmalonyl–CoA epimerase	0.51	0.40	0.51	0.42	0.51
GE00217	*meaA*	Ethylmalonyl–CoA mutase	**1.43**	0.81	1.04	**1.26**	1.08
GE01348	*yngJ*	(2S)-Methylsuccinyl–CoA dehydrogenase	**1.61**	0.63	1.10	1.16	1.08
GE00952	*mch*	2-Methylfumaryl–CoA hydratase	**2.08**	0.79	**1.52**	**1.74**	**1.48**
GE03891	*mcl*	Malyl–CoA lyase	**1.56**	0.55	1.14	1.03	1.00
GE00948	*mcl2*	(3S)-malyl–CoA thioesterase	0.93	0.34	0.21	0.65	0.61
GE03545	*pccA*	Propionyl–CoA carboxylase alpha chain	0.96	0.75	1.30	0.84	0.94
GE03546	*pccB*	Propionyl–CoA carboxylase beta chain	**1.99**	1.20	**2.53**	**1.46**	**1.53**
GE03765			0.21	0.92	**2.00**	**1.80**	**1.68**
GE03541	*mutB*	Methylmalonyl–CoA mutase	1.13	1.20	1.24	1.17	**1.20**
GE03543	*mutA*		0.80	0.71	0.95	0.73	0.81
GE05659	*bhbA*		0.43	0.67	0.51	0.52	0.59
GE05660	*meaA*		0.57	0.69	0.42	0.45	0.57
GE05792	*scpA*		0.55	0.68	0.29	0.27	0.25
GE05793	*bhbA*		0.77	0.67	0.34	0.32	0.31

^a^ Fold change ≥ 1.2 with FDR and *p* value < 0.05 are indicated in boldface. ^b^ The meaning of each sample compared with citrate (T0C): T0D, strain YN2 grown on dioxane when half of the dioxane degraded; T1C, 24 h after carbon source switched from citrate to dioxane; T2C, 48 h after carbon source switched from citrate to dioxane; T1D, strain YN2 grown on dioxane when dioxane depleted; T2D, strain YN2 grown on dioxane when TOC ceased decreasing.

## Data Availability

Publicly available datasets were analyzed in this study. These data can be found at https://www.ncbi.nlm.nih.gov/ (accessed on 22 August 2021) in GenBank, accession numbers CP063362-CP063366, and in SRA database, bioproject ID: PRJNA640796.

## Supplementary Materials

The following are available online at https://www.mdpi.com/article/10.3390/ijms221910435/s1, Figure S1: Correlation between RT-qPCR and RNA-seq, Table S1: Primers used in RT-qPCR for validation, Table S2: DEGs of each relationship between T0C-vs-T0D, T0C-vs-T1C, and T0C-vs-T2C, Table S3: Upregulated DEGs of different degradation stages, Table S4: Expression of quorum-sensing-related genes, Table S5: Expression of genes encoding transporters, Table S6: Expression of Genes encoding two-component system, Table S7: Expression of genes related to chemotaxis, Table S8: DEGs with FPKM higher than 100.
