# Supplementary material for: Adaption and Degradation Strategies of Methylotrophic 1,4-Dioxane Degrading Strain Xanthobacter sp. YN2 Revealed by Transcriptome-Scale Analysis
Source: Int J Mol Sci. 2021 Sep 28;22(19):10435. doi: 10.3390/ijms221910435 (PMC8508750; doi:10.3390/ijms221910435)
Supplement: Supplementary file 1 [file ijms-22-10435-s001.zip › Table S1 Primers used in RT-qPCR for validation.pdf]

Table S1 Primers used in quantitative real-time reverse transcriptase-PCR

Primers	Sequences
thmA-F	5′-CGATACAGCCAGAGACAT-3′
thmA-R	5′-CGAACGGATGGAGTAGAT-3′
thmB-F	5′-CGAAGATACCGACGACAT-3′
thmB-R	5′-CCTTGCCGTTGAAATAGAC-3′
thmC-F	5′-CGAAGGTGAAGAAGTGAT-3′
thmC-R	5′-GTCTGCGAGGAGTATTTC-3′
thmD-F	5′-AAGACATCATCAACGCCGTG-3′
thmD-R	5′-TGCTCTTCTCGGTCAGACAG-3′
thmE-F	5′-CGTCACCTACAAGTTCCACA-3′
thmE-R	5′-CGGAAGAAACAAGCGGCGAG-3′
thmF-F	5′-CTGCTCCGCCGTCGTAAC-3′
thmF-R	5′-TGGAAGATCAGGTCGACAGA-3′
GE06014-F	5′-AAGGTGCCGTCTATGATG-3′
GE06014-R	5′-ACTGTCGTCTTGGTGATG-3′
GE05990-F	5′-GCCACATTCCACAGTCTT-3′
GE05990-R	5′-CAAGCGACGGTTCTCAAG-3′
GE02965-F	5′-CTTGCGGATGGCTTCTAC-3′
GE02965-R	5′-CCTCTTCCTCGTCCTTGA-3′
GE02036-F	5′-ACGCTCTCATGCAACCTG-3′
GE02036-R	5′-AGGGCCATCATTCCGGTA-3′
GE05991-F	5′-AGTTTTACCGGCTCGACA-3′
GE05991-R	5′-TTCTTGCCGCCCATCTCG-3′
GE05994-F	5′-AACACCCGGATATCGACA-3′
GE05994-R	5′-TCACCCGCTTCATCGTTT-3′
GE02333-F	5′-AAGTTCGCCGCCAATCTC-3′
GE02333-R	5′-CCAGCACTTCCTTGTCGTAG-3′
GE05988-F	5′-GGTTCTGCTTGTGATGAG-3′
GE05988-R	5′-TTGGTGATGCCGAGATTA-3′
16S-F	5′-TGATAAGCCGAGAGGAAGG-3′
16S-R	5′-CTGTCACCGCCATTGTAG-3′
